# Unmasking Mucopolysaccharidosis Type I in a Patient With Wolf–Hirschhorn Syndrome: Diagnostic Overshadowing

**DOI:** 10.1002/jmd2.70117

**Published:** 2026-09-06

**Authors:** Karla Cifuentes‐Uribe, Sandrine Girard, Roseline Froissart, Magali Pettazzoni, Nathalie Guffon

**Affiliations:** ^1^ Reference Center for Inherited Metabolic Disorders, Femme Mère Enfant Hospital Hospices Civils de Lyon Bron France; ^2^ Laboratory of Hematology, Center of Biology and Pathology East Hospices Civils de Lyon Lyon France; ^3^ Department of Biochemistry and Molecular Biology, Inborn Error of Metabolism Unit Hospices Civils de Lyon Lyon France

**Keywords:** diagnostic overshadowing, dual diagnosis, gasser cells, lysosomal storage disorder, Mucopolysaccharidosis Type I, Wolf–Hirschhorn syndrome

## Abstract

Mucopolysaccharidosis Type I (MPS I) is a rare lysosomal storage disorder caused by α‐l‐iduronidase deficiency, leading to glycosaminoglycan accumulation and multisystem involvement. Wolf–Hirschhorn Syndrome (WHS) is a chromosomal disorder characterized by growth delay, dysmorphism, and developmental impairment. Because the *IDUA* gene is located within the 4p16.3 region, the coexistence of WHS and MPS I is biologically plausible but rarely documented. We describe a 2‐year‐old child of North African origin presenting with dysmorphic features, developmental delay, and thoracolumbar kyphosis. Chromosomal microarray analysis identified a 5.8 Mb deletion at 4p16.3–p16.2, confirming WHS at 17 months. However, additional clinical features remained unexplained. Incidentally, during a routine blood test, a blood smear revealed lymphocytes with metachromatic inclusions, raising suspicion of a lysosomal storage disorder. An enzymatic assay confirmed α‐l‐iduronidase deficiency, and molecular analysis identified biallelic pathogenic alterations in the *IDUA* gene located on chromosome 4p16.3, establishing the diagnosis of MPS I at 18 months. Enzyme replacement therapy was initiated at 19 months and was associated with stabilization of the somatic manifestations of MPS I, including improvement in respiratory symptoms. This observation highlights the risk of diagnostic overshadowing in rare diseases. A confirmed genetic diagnosis should not preclude further investigations when clinical features are discordant.

## Key Points

- Diagnosis overshadowing may delay the recognition of treatable inherited metabolic diseases.
- Discordant clinical features should prompt further investigations despite an established genetic diagnosis.
- Routine peripheral blood smear examination can provide an unexpected clue leading to diagnosis of MPS I.

## 1 Introduction

Wolf–Hirschhorn syndrome (WHS) is a rare chromosomal disorder due to a deletion on chromosome 4p, characterized by growth retardation, intellectual disability, and distinctive craniofacial features [1, 2]. The coexistence of multiple genetic disorders in a single patient may complicate clinical interpretation. We describe a case illustrating diagnostic overshadowing, where a metabolic disorder remained unrecognized despite a confirmed chromosomal diagnosis.

## 2 Clinical Description

**FIGURE 1:**
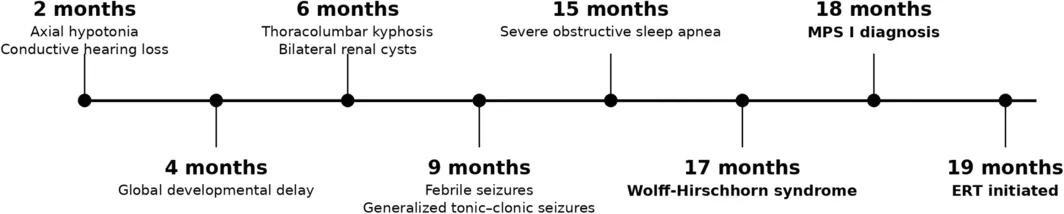
Diagnostic timeline highlighting early clinical features, initial diagnosis of Wolf‐Hirschhorn syndrome, and subsequent identification of MPS I, illustrating diagnostic overshadowing. ERT: enzyme replacement therapy.

**FIGURE 2:**
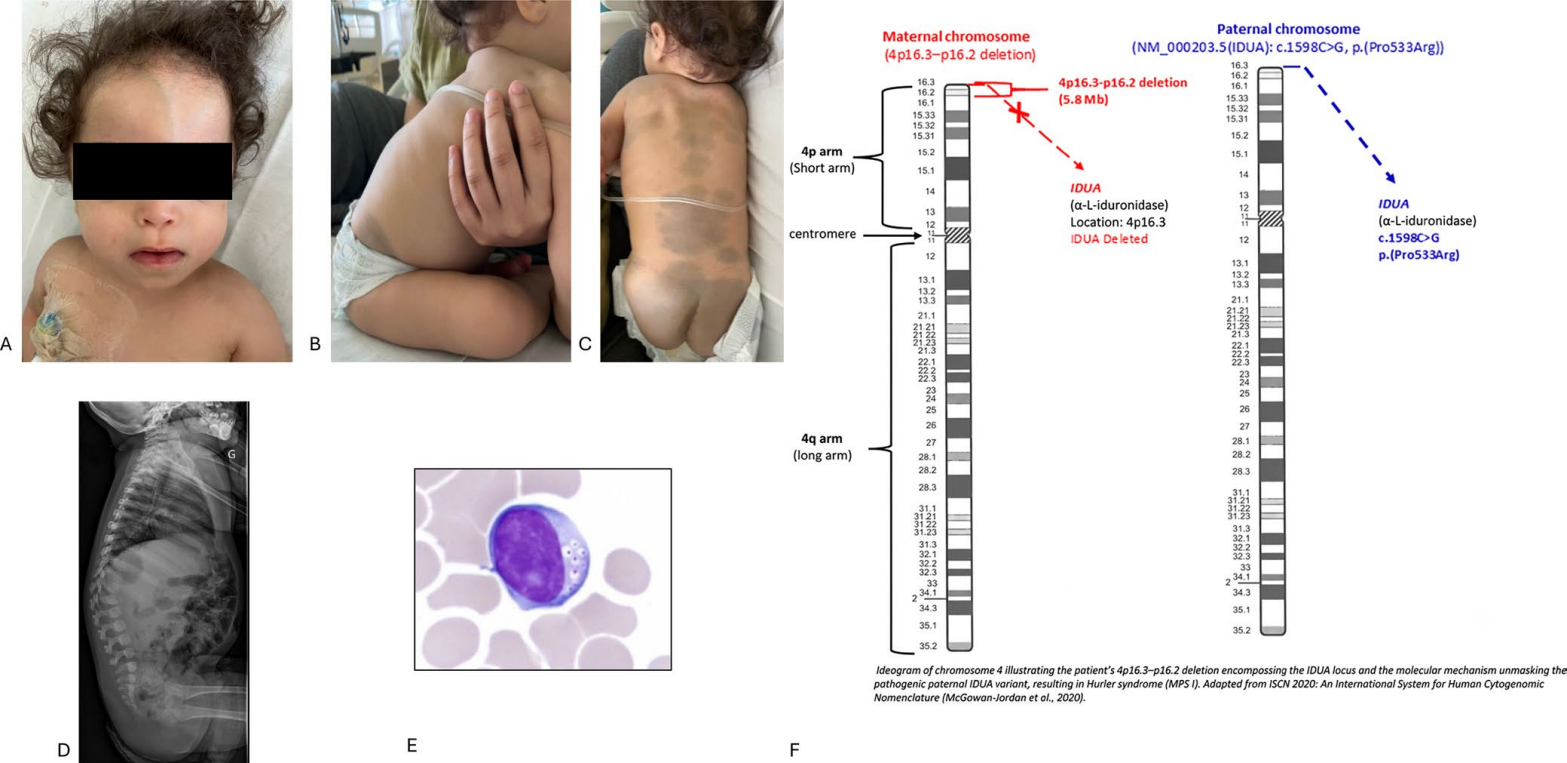
Clinical, radiological, cytological, and molecular features of the patient. (A) Dysmorphic facial characteristics, (B) thoracolumbar kyphosis, (C) extensive dermal melanocytosis, (D) lateral spine radiograph obtained after the diagnosis of MPS I, showing characteristic anterior vertebral beaking consistent with dysostosis multiplex. (E) Peripheral blood smear showing a Gasser cell, (F) chromosome 4 ideogram illustrating the 4p16.3–p16.2 deletion encompassing *IDUA* and the molecular mechanism underlying the dual diagnosis. Images are published with parentally informed consent.

The patient is a 2‐year‐old child, third‐born to nonconsanguineous parents of North African origin, with no family history of genetic disease. Early clinical manifestations included extensive congenital dermal melanocytosis, axial hypotonia, soft cleft palate, feeding difficulties, and conductive hearing loss. Echocardiography revealed a small patent foramen ovale and a small patent ductus arteriosus. Developmental delay became apparent within the first months of life, associated with progressive thoracolumbar kyphosis and febrile seizures. During follow‐up, recurrent respiratory infections and obstructive sleep apnea also emerged. Initial craniofacial features were consistent with WHS, including hypertelorism, a broad flat nasal bridge, a short nose, micrognathia, and a prominent glabella. During the second year of life, progressive coarsening of the facial features, with fuller eyebrows, prominent lips, and a depressed nasal bridge, became apparent. The chronological progression of clinical features and diagnostic steps is summarized in Figure 1. At 17 months, chromosomal microarray analysis identified a 5.8 Mb deletion at 4p16.3–p16.2, consistent with WHS [2]. Although some clinical features remained not fully explained, no specific additional investigations were initially directed toward a metabolic disorder. At 18 months, a “Blasts” flag generated by the automated hematology analyzer prompted mandatory manual peripheral blood smear review. Microscopic examination revealed lymphocytes containing metachromatic cytoplasmic inclusions (Gasser cells) rather than true blasts, raising suspicion of a lysosomal storage disorder [3, 4]. This incidental finding prompted further targeted investigations. Urinary glycosaminoglycans were elevated with abnormal presence of dermatan and heparan sulfate, and enzymatic analysis demonstrated deficient α‐l‐iduronidase activity in leukocytes, establishing the diagnosis of Mucopolysaccharidosis Type I (MPS I) [5, 6]. Molecular analysis confirmed a biallelic pathogenic mechanism in the *IDUA* gene, consisting of a complete deletion of one allele and a pathogenic variant on the remaining allele, c.1598C>G (p.Pro533Arg) (inherited from the father). Representative clinical, radiological, cytological, and molecular findings are presented in Figure 2. The lateral spine radiograph, obtained after the diagnosis of MPS I, demonstrates characteristic anterior vertebral beaking. Enzyme replacement therapy (ERT) with ALDURAZYME (laronidase), a recombinant form of human α‐l‐iduronidase, was initiated at 19 months [7]. Weekly infusions were well tolerated, with no infusion‐associated reactions. During follow‐up, upper respiratory tract infections markedly decreased, allowing progressive discontinuation of noninvasive ventilation. Cardiac disease remained stable, with mild aortic valve thickening and Grade 1 aortic regurgitation. Thoracolumbar kyphosis and corneal clouding remained stable. Joint involvement was limited to mild stiffness of the shoulders and knees. Neurologically, hypotonia persisted, but motor development progressed (developmental age approximately 9 months at a chronological age of 2 years and 9 months), while epilepsy remained well controlled with levetiracetam. Urinary glycosaminoglycan excretion decreased to the upper limit of the age‐adjusted reference range.

## 3 Discussion

**FIGURE 3:**
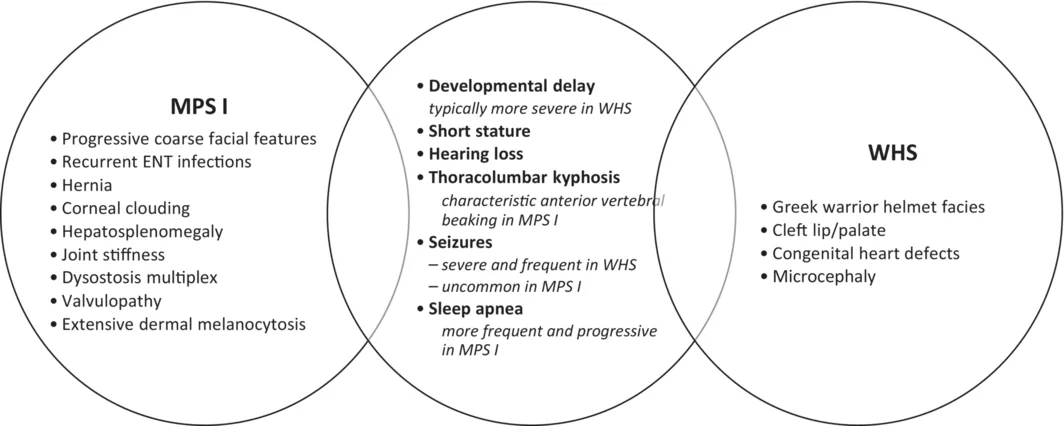
Shared and distinguishing clinical features of Wolf–Hirschhorn syndrome and Mucopolysaccharidosis Type I. Clinical manifestations in the overlapping area may occur in both disorders but differ in frequency, severity, or associated characteristics.

This case highlights the diagnostic complexity associated with overlapping genetic conditions and underscores the importance of maintaining diagnostic vigilance, even after an initial molecular diagnosis has been established. The patient was first diagnosed with WHS, a condition explaining several early clinical features [8, 9]. However, the persistence and progression of additional manifestations—particularly, thoracolumbar kyphosis, extensive dermal melanocytosis, and worsening respiratory involvement—suggested an incomplete phenotypic explanation, although these findings did not initially prompt targeted investigations for a metabolic disorder [6, 7]. A comparative overview of clinical features associated with MPS I and WHS (Figure 3) highlights both overlapping manifestations and distinguishing features. While global developmental delay, short stature, and hearing loss may be observed in both conditions [10, 11], other features such as extensive dermal melanocytosis, progressive kyphosis with anterior vertebral beaking, and respiratory involvement are more suggestive of MPS I [6, 11, 12, 13]. In this case, the diagnosis of MPS I was not driven by strong clinical suspicion but rather by the incidental identification of metachromatic inclusions in lymphocytes (Gasser cells) on a peripheral blood smear, a nonspecific marker of lysosomal glycosaminoglycan storage reported in several MPS subtypes, including MPS VI [3, 4]. This finding prompted further investigations, including urinary glycosaminoglycan analysis and α‐l‐iduronidase enzymatic assay, which ultimately established the diagnosis of MPS I. This case illustrates how a non‐specific but readily identifiable cytological finding can trigger targeted biochemical investigations, leading to a precise diagnosis. These findings also highlight the continuing diagnostic value of routine peripheral blood smear examination, even in the era of advanced genomic testing, as unexpected cytological findings may reveal treatable inherited metabolic disorders. This situation illustrates a period of diagnostic overshadowing, during which the initial genetic diagnosis may have contributed to delaying the recognition of a second condition. It also emphasizes that routine investigations, when carefully interpreted, may reveal unexpected diagnoses and should not be overlooked in complex clinical settings. From a clinical perspective, early identification of MPS I is crucial, as timely initiation of ERT or hematopoietic stem cell transplantation (HSCT) can significantly impact disease progression [14, 15]. Based on the early clinical presentation and molecular findings, the patient fulfilled the clinical and biochemical criteria for severe MPS I (Hurler syndrome). Although HSCT is the standard of care for eligible patients with Hurler syndrome when performed early, it was not considered appropriate in this case because of the severe neurodevelopmental impairment already present at diagnosis due to WHS and MPS I. Following multidisciplinary discussion, ERT was therefore considered the most appropriate therapeutic option and initiated shortly after diagnosis. Although neurological outcome remained largely determined by the underlying WHS, ERT was associated with stabilization of the somatic manifestations of MPS I and improvement of respiratory symptoms. This case also underscores the distinction between phenotypic expansion and dual diagnosis. While phenotypic variability is well described in both MPS I and WHS [2, 10, 16, 17], attributing all clinical manifestations to a single condition may lead to misinterpretation. The persistence of unexplained or atypical features should prompt clinicians to reconsider the diagnostic hypothesis rather than expand the known phenotype of the initial disorder without sufficient evidence. From a molecular standpoint, this case provides an instructive example of unmasking of an autosomal recessive disorder by a chromosomal deletion. The *IDUA* gene is located on chromosome 4p16.3, within the region affected by the deletion responsible for WHS [2]. Molecular analysis confirmed a biallelic pathogenic mechanism, consisting of a deletion of one allele and a pathogenic variant on the remaining allele. This configuration illustrates the mechanism of unmasking of an autosomal recessive disorder by a chromosomal deletion. The variant p.Arg533Pro is a well‐established pathogenic variant associated with MPS I and is classified as pathogenic according to ACMG criteria. It has been reported in numerous MPS I patients and is particularly frequent in North African populations, especially among individuals of Moroccan ancestry, where it represents one of the most prevalent disease‐causing *IDUA* variants [18]. The hemizygous state resulting from the 4p16.3 deletion unmasked this pathogenic allele and explains the profound α‐l‐iduronidase deficiency observed in our patient and the severe phenotype. This case highlights the importance of considering dual molecular diagnoses in patients with atypical clinical evolution, even when an initial genetic diagnosis has been established. Chromosomal deletions may not only directly cause disease but also reveal underlying recessive conditions. It also emphasizes the need to consider contiguous gene deletions as a potential mechanism for unmasking autosomal recessive disorders.

## 4 Conclusion

Identifying one genetic diagnosis should not preclude the search for another when the phenotype remains incomplete. In the era of genomic medicine, diagnostic vigilance is essential to avoid missed opportunities for early treatment. Prompt recognition of conditions such as MPS I can significantly alter disease trajectory and patient prognosis. This case illustrates that a confirmed genetic diagnosis should not end the diagnostic journey when the clinical picture remains unresolved.

## Funding

The authors have nothing to report.

## Conflicts of Interest

The authors declare no conflicts of interest.

## Data Availability

Data sharing not applicable to this article as no datasets were generated or analysed during the current study.

## References

[1] A. Battaglia , J. C. Carey , and S. T. South , “Wolf‐Hirschhorn Syndrome: A Review and Update,” American Journal of Medical Genetics Part C: Seminars in Medical Genetics 169, no. 3 (2015): 216–223, 10.1002/ajmg.c.31449. 26239400

[2] A. Battaglia and J. C. Carey , “The Delineation of the Wolf‐Hirschhorn Syndrome Over Six Decades: Illustration of the Ongoing Advances in Phenotype Analysis and Cytogenomic Technology,” American Journal of Medical Genetics Part A 185, no. 9 (2021): 2748–2755, 10.1002/ajmg.a.62341. 34002939

[3] M. A. W. de Melo , M. S. Kertenetzky , C. M. da Silveira , et al., “Gasser Cell: A Biomarker of Response to Enzyme Replacement Therapy in Patients With Mucopolysaccharidosis Type VI,” Research, Society and Development 10, no. 5 (2021): e24510514726, 10.33448/rsd-v10i5.14726.

[4] R. Jain , U. Khurana , B. D. Bhan , G. Goel , and N. Kapoor , “Mucopolysaccharidosis: A Case Report Highlighting Hematological Aspects of the Disease,” Journal of Laboratory Physicians 11, no. 1 (2019): 97–99, 10.4103/JLP.JLP_126_18. 30983812 PMC6437814

[5] L. A. Clarke , R. Giugliani , N. Guffon , et al., “Genotype‐Phenotype Relationships in Mucopolysaccharidosis Type I (MPS I): Insights From the International MPS I Registry,” Clinical Genetics 96, no. 4 (2019): 281–289, 10.1111/cge.13583. 31194252 PMC6852151

[6] F. Kubaski , F. de Oliveira Poswar , K. Michelin‐Tirelli , et al., “Mucopolysaccharidosis Type I,” Diagnostics 10, no. 3 (2020): 161, 10.3390/diagnostics10030161. 32188113 PMC7151028

[7] C. S. Hampe , J. Wesley , T. C. Lund , et al., “Mucopolysaccharidosis Type I: Current Treatments, Limitations and Prospects for Improvement,” Biomolecules 11, no. 2 (2021): 189, 10.3390/biom11020189. 33572941 PMC7911293

[8] A. Battaglia , T. Filippi , and J. C. Carey , “Update on the Clinical Features and Natural History of Wolf‐Hirschhorn (4p‐) Syndrome: Experience With 87 Patients and Recommendations for Routine Health Supervision,” American Journal of Medical Genetics ‐ Seminars in Medical Genetics, Part C 148, no. 4 (2008): 246–251, 10.1002/ajmg.c.30187. 18932224

[9] J. Paprocka , K. Kaminiów , O. Yetkin , et al., “Clinical and Epilepsy Characteristics in Wolf‐Hirschhorn Syndrome (4p‐): A Review,” Seizure 114 (2024): 14–23, 10.1016/j.seizure.2022.12.001. 36526544

[10] A. Battaglia , A. Lortz , and J. C. Carey , “Natural History Study of Adults With Wolf–Hirschhorn Syndrome 1: Case Series of Personally Observed 35 Individuals,” American Journal of Medical Genetics. Part A 185, no. 6 (2021): 1794–1802, 10.1002/ajmg.a.62176. 33760347

[11] C. S. Hampe , J. B. Eisengart , T. C. Lund , et al., “Mucopolysaccharidosis Type I: A Review of the Natural History and Molecular Pathology,” Cells 9, no. 8 (2020): 1838, 10.3390/cells9081838. 32764324 PMC7463646

[12] S. Athanti , S. Mouzam , and M. Sohail Ahmed , “Atypical Mongolian Spots With Hurler's Disease: A Case Report,” Cureus 16, no. 4 (2024): e58501, 10.7759/cureus.58501. 38765368 PMC11101911

[13] S. J. Mao , Y. M. Zu , T. M. Yuan , and C. Zou , “The Significance of Mongolian Spots for Primary Screening of Mucopolysaccharidosis,” Research Square (2023), 10.21203/rs.3.rs-3119136/v1.

[14] N. Guffon , M. Pettazzoni , N. Pangaud , et al., “Long‐Term Disease Burden Post‐Transplantation: Three Decades of Observations in 25 Hurler Patients Successfully Treated With Hematopoietic Stem Cell Transplantation (HSCT),” Orphanet Journal of Rare Diseases 16 (2021): 60, 10.1186/s13023-020-01644-w. 33517895 PMC7847591

[15] N. Guffon , M. Pettazzoni , N. Pangaud , et al., “Clinical Outcomes of Exclusive Enzyme Therapy (Laronidase) in a Cohort of Patients With Mucopolysaccharidosis Type I,” Orphanet Journal of Rare Diseases 21 (2026): 11, 10.1186/s13023-025-04157-6. PMC12797645 41353341

[16] C. Chaudhry , A. Kaur , I. Panigrahi , and A. Kaur , “Wolf–Hirschhorn Syndrome: A Case Series From India,” American Journal of Medical Genetics ‐ Seminars in Medical Genetics, Part A 182, no. 12 (2020): 3048–3051, 10.1002/ajmg.a.61856. 32914558

[17] C. Xia , D. Kumar , B. You , et al., “Wolf‐Hirschhorn Syndrome With Hyperparathyroidism: A Case Report and a Narrative Review of the Literature,” Journal of Pediatric Genetics 12, no. 4 (2023): 312–317, 10.1055/s-0041-1729751. 38162156 PMC10756731

[18] E. Poletto , G. Pasqualim , R. Giugliani , U. Matte , and G. Baldo , “Worldwide Distribution of Common IDUA Pathogenic Variants,” Clinical Genetics 94, no. 1 (2018): 95–102, 10.1111/cge.13224. 29393969

